# Quantitative proteomics profiling reveals activation of mTOR pathway in trastuzumab resistance

**DOI:** 10.18632/oncotarget.17415

**Published:** 2017-04-25

**Authors:** Wenhu Liu, Jinxia Chang, Mingwei Liu, Jiangbei Yuan, Jinqiang Zhang, Jun Qin, Xuefeng Xia, Yi Wang

**Affiliations:** ^1^ School of Pharmaceutical Sciences and Innovative Drug Research Center, Chongqing University, Chongqing 401331, China; ^2^ State Key Laboratory of Proteomics, National Center for Protein Sciences, Beijing Proteome Research Center, Beijing 102206, China; ^3^ School of Pharmacy, North Sichuan Medical College, Nanchong 637007, China; ^4^ School of Basic Medical Sciences, North Sichuan Medical College, Nanchong 637007, China; ^5^ Verna and Marrs McLean Department of Biochemistry and Molecular Biology, Baylor College of Medicine, Houston, Texas 77030, USA; ^6^ Department of Molecular and Cellular Biology, Baylor College of Medicine, Houston, Texas 77030, USA

**Keywords:** HER2, trastuzumab resistance, mTOR signaling pathway, gastric cancer, LC-MS/MS

## Abstract

Trastuzumab is an antibody-based therapy drug targeting HER2-overexpressing tumors. While it has been proven to be very successful initially, most patients eventually develop resistance to trastuzumab. The mechanism of drug resistance is not well understood. Identifying pathways that mediate trastuzumab resistance will improve our understanding of the underlying mechanism and is crucial for the development of therapeutic strategies to overcome resistance.

Here we report a quantitative proteomics profiling of a trastuzumab-sensitive (T-S) gastric cancer cell line NCI N87 and a trastuzumab-resistant NCI N87 (T-R) subline generated by low-dose, continuous trastuzumab treatment. By identifying proteins differentially expressed in these two cell lines, we show that multiple pathways including mTOR, Wnt, DNA damage response and metabolic pathways are significantly altered. We further confirm by western blotting that protein levels of multiple components of the mTOR pathway, including mTOR, AKT and RPS6KB1, are increased, whereas AKT1S1 is decreased, suggesting the activation of mTOR pathway. Importantly, treatment of AZD8055, an mTOR inhibitor, leads to the decreased phosphorylation levels of mTOR downstream molecules RPS6KB1 at Thr421/Ser424 and AKT at Ser473. Furthermore, AZD8055 also preferentially reduces viability, and inhibits migration and invasion abilities of the T-R cells. Together, our findings indicate that mTOR pathway is among multiple signaling pathways that mediate trastuzumab resistance in NCI N87 T-R cells, and that mTOR inhibitors may be used to treat trastuzumab resistant, HER2-positive gastric cancer tumors.

## INTRODUCTION

Globally, gastric cancer (GC) is the fifth most common malignancy and the third leading cause of cancer related death [1]. GC is often detected at late stages with few treatment options. One actionable GC biomarker is human epidermal growth factor receptor 2 (HER2 or ErbB2), which is encoded by the *ERBB2* gene [2, 3]. It is a member of the HER family proteins and its overexpression has a positive correlation with tumor cell proliferation, adhesion, migration and invasion [4]. Approximately 15∼20% of GC cases have *ERBB2* DNA amplification along with consistent overexpression of HER2 protein [5, 6]. The aberrant overexpression or activation of HER2 is thought to trigger multiple cellular signaling pathways which drive abnormal cell proliferation, drug resistance and metastasis [4, 7].

Molecular targeting therapy has been deemed a highly effective strategy for cancer treatment. Trastuzumab (Herceptin^®^), a humanized monoclonal antibody against the extracellular domain of HER2, has been widely used in HER2 positive breast cancer (BC) and GC in combination with chemotherapy in clinical treatment [8–11]. However, due to the acquired resistance to trastuzumab, the effect is limited. It was shown that only less than 13% of the patients benefitted from the trastuzumab therapy [12, 13].

Several pathways for trastuzumab resistance in GC have been identified. Some genetic mutations may contribute to GC survival independent of the therapeutic targets. For example, the p110a subunit of PI3K (PIK3CA) and c.428T>C (p.V143A) homozygous mutation in exon5 of *TP53* gene lead to drug resistance and therefore potentially affect the efficacy of clinical therapy [14, 15]. Activation of HER2 target mutation, up-regulation of the PI3K signaling pathway, accumulation of truncated HER2 receptor, activation of insulin-like growth factor receptor (IGFR) and loss of the PTEN, are among the major pathways identified in BC [16–21]. Additionally, activation of crosstalk of HER2 to other molecules such as HER3 and MET leads to subsequent activation of downstream signaling pathways [10, 22, 23]. Activation of alternative pathways, such as amplification or mutation of c-MET and SRC activation, low immune response [17, 24], and overexpression of Cyclin E have also been shown in BC [25]. While some of these pathway alterations are shared by GC, there are also GC-specific mechanisms. Activation of the IL-6/STAT3/Jagged-1/Notch pathways [26], overexpression of FGFR3 and its ligand FGF9 [27], catecholamine-induced β2-adrenergic receptor activation which mediates desensitization by upregulating MUC4 expression [28], activation of STAT3 via upregulation of MUC1 and MUC4 expression [29], are some examples. Comparing to BC, molecular pathways that mediate acquired trastuzumab-resistance in GC is less understood [30–32].

While DNA sequencing has been a method of choice in the past to identify activated oncogenic pathways in tumors at genomic level, global proteome profiling by mass spectrometry (MS) has emerged as a powerful tool to characterize proteomics changes [15, 33]. Our lab has developed a fast sequencing (Fast-seq) and a label-free quantification (LFQ) workflow (Fast-quan), by which more than 8,000 proteins can be identified and quantified within 12 hours of MS running time [34, 35]. This workflow allows us to analyze a variety of biological samples with consistent results.

In this study, we performed proteomic profiling of a pair of gastric cancer cell lines consisting of a trastuzumab-sensitive NCI N87 and a trastuzumab-resistant subline derived from NCI N87. We identified differentially expressed proteins and investigated the corresponding signaling pathways by bioinformatics analysis. Additional biochemical and functional validation suggest that the mTOR pathway is activated in T-R cells, implicating the mTOR pathway as a potential molecular target for treating tumors arising from trastuzumab resistance.

## RESULTS

### Proteomic profiling of NCI N87 T-S and T-R cells

We obtained a pair of T-S and T-R cells as described in [26] from the Shi lab. The T-R cells exhibited marked resistance to trastuzumab compared with the T-S cells (Supplementary Figure 1). Western blotting showed that the HER2 levels in the 2 cell lines were comparable, which was in accordance with the MS data (Supplementary Figure 2A, 2B and 2C). Additionally, T-R cells grew faster than T-S cells (Supplementary Figure 3A), and displayed typical morphology of epithelial to mesenchymal transition (EMT) (Supplementary Figure 3B), implying their higher invasive and metastatic potentials. All these characteristics are consistent with the previous report [26].

**Figure 1:**
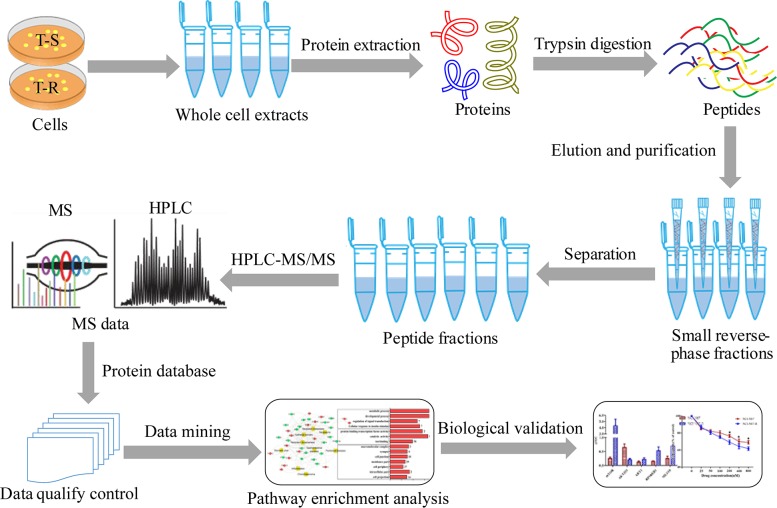
A schematic diagram of experimental workflow To exploit underlying mechanisms of trastuzumab-resistance in gastric cancer cells with HER2 amplification, we employed quantitative proteomics to profile global proteomes of both T-S and T-R cells. The processing and sampling of the whole cellular extracts were accomplished following our protocols.

**Figure 2:**
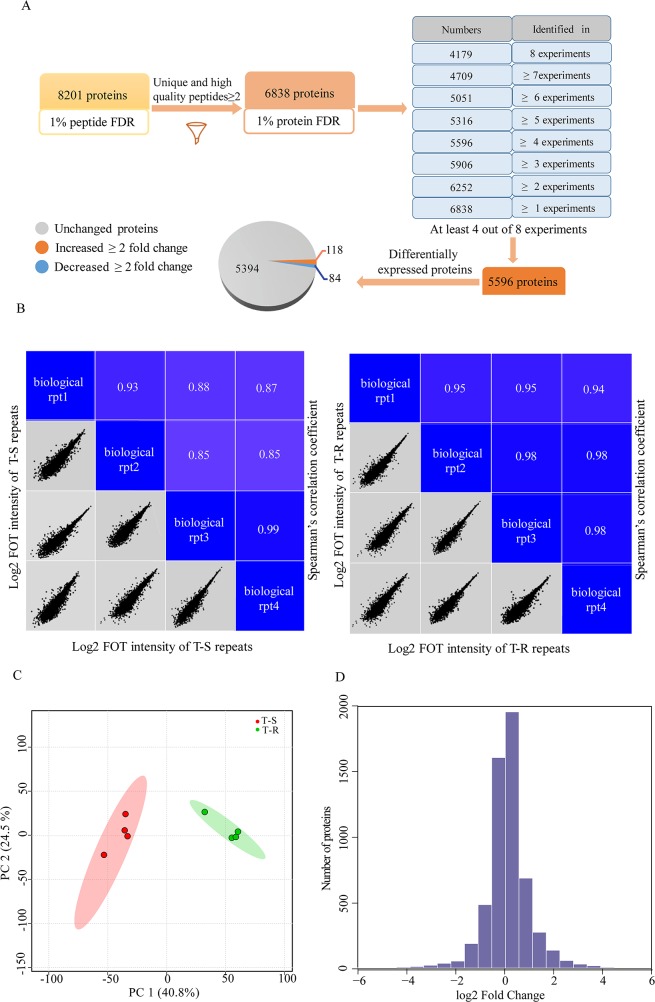
A workflow of the whole cellular extracts and identification of differentially expressed proteins **(A)** A workflow for data filtering and processing. **(B)** Spearman's correlation coefficients were calculated to assess the experiment reproducibility. The lower-left half shows pairwise scatter plots of four biological repeats, with x and y axes representing log2 FOT intensity. The upper-right half shows pairwise Spearman's correlation coefficients for the same comparison. **(C)** PCA analysis was performed to distinguish T-S cells from T-R cells. **(D)** The distribution of protein abundance ratios is displayed with histogram. The fold changes of T-R cells/T-S cells are shown in log2 scale on the x axis and the number of proteins are shown on the y axis.

To identify signal transduction pathways that mediate trastuzumab resistance, we performed quantitative proteomic profiling of the T-S and T-R cells. As shown in Figure 1, we performed 4 biological replicates on both cell lines and quantified protein abundance with label-free, intensity-based absolute quantification (iBAQ). We normalized protein loading by taking the fraction of total (FOT) followed by multiplication of 10^5^ to obtain iFOT5. A total of 8201 proteins that were detected with at least 2 unique and high quality peptides (1% false discovery rate (FDR) at the peptide level and Mascot ion score greater than 20) were identified. Among them, 6838 proteins were identified at 1% protein FDR (Figure 2A, Supplementary Table 1), and 5596 were found reproducibly in at least 4 of the 8 experiments (Figure 2A, Supplementary Table 2). These 5596 proteins were used for subsequent statistical and bioinformatics analyses. The high correlation (*r* >0.8) of each pair of the 4 replicates demonstrated a good reproducibility of our measurements (Figure 2B). A principal component analysis (PCA) showed that the T-S and T-R data sets were well separated, and four biological replicates of each cell line were well-clustered (Figure 2C). The distribution of fold changes was shown in a histogram (Figure 2D).

### Enrichment analysis of differentially expressed proteins and pathways

**Figure 3:**
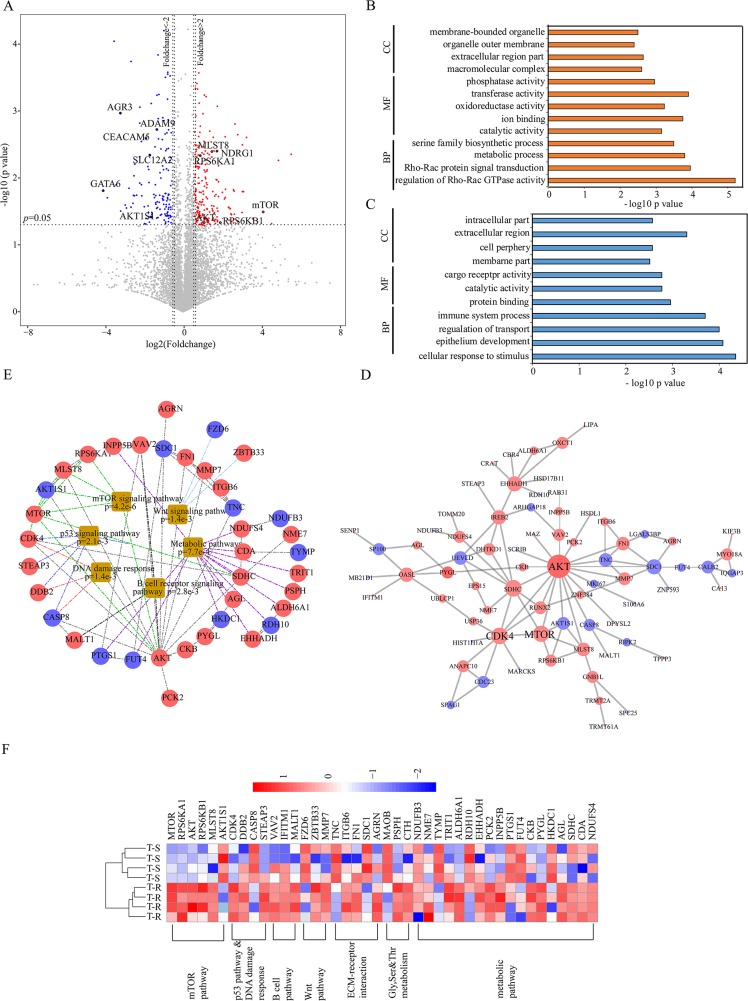
Bioinformatics analysis of differentially expressed proteins and pathways **(A)** Differential proteins in T-R cells and T-S cells are illustrated in volcano plot. The mean ratio of four biological repeats (T-R cells/T-S cells) was plotted in log2 scale (x axis) against the corresponding –log10 *p* value (y axis). The vertical dotted lines mark twofold change and horizontal dotted line represent cutoff *p* value = 0.05. Proteins which showed fold changes greater than 2 or less than 0.5 and *p*<0.05 were considered up- or down-regulated and marked in red and blue respectively. Some proteins were emphasized by enclosed circle. The gray dots were considered as no significant change. **(B)** Gene ontology enrichment analysis of up-regulated proteins (red bar) and **(C)** down-regulated proteins (blue bar) involved in cell component (CC), molecular function (MF) and biological process (BP). **(D)** Network analysis of differential proteins were done with Cytoscape software. Red nodes represent increased proteins and blue nodes represent decreased ones. Size of nodes correspond to numbers of interacting neighbors. **(E)** KEGG analysis identified major biological pathways in which the differential proteins are involved. Each colored line indicated different pathways. **(F)** Heat map visualization of 42 differential proteins identified from several pathways in both cell lines. The increased and decreased proteins are indicated by range of red and blue intensities, respectively.

We performed student's t tests to identify differentially expressed proteins that were statistically significant. As illustrated in the volcano plot (Figure 3A, Supplementary Table 3), the abundance of 118 proteins (2.1% of the proteome) showed more than twofold increase in T-R cells (*p* value <0.05, marked as red dots), whereas 84 proteins (1.5% of the proteome) showed more than twofold decrease in T-R cells (*p* value <0.05, marked as blue dots). The remaining 5394 proteins (96.4% of the proteome) were considered as not significantly changed. Seven differentially expressed proteins that were identified with 1-3 spectrum counts (PSMs) were manually validated (Supplementary Figure 4). The differentially expressed proteins were analyzed by gene ontology (GO) terms enrichment analysis using the WEB-based Gene Set Analysis Toolkit (http://bioinfo.vanderbilt.edu/webgestalt/option.php) [36]. The enrichment of increased or decreased proteins in cellular component, biological process and molecular function are shown in Figure 3B and 3C, respectively. Proteins that are increased in T-R cells are annotated as localized in extracellular region, macromolecular complex, and membrane-bounded organelle, whereas those decreased in T-R cells are annotated as residing in extracellular region, intracellular part and cell periphery. As for biological processes, increased proteins include those involved in regulation of Rho-Rac GTPase activity, Rho-Rac protein signal transduction and metabolic process, and decreased proteins were primarily shown as cellular response to stimuli, epithelial development and regulation of transport. As to molecular functions, the increased proteins are enriched in transferase activity, ion binding and phosphatase activity, whereas the decreased proteins function in protein binding, catalytic activity and cargo receptor activity. We employed the STRING database (http://string.embl.de/) [37] to build a network of the differentially expressed proteins using high confidence (scores >0.7) expressions and visualize the resulting network using Cytoscape (v3.4.0). We found that AKT, mTOR and CDK4 represent three main hubs formed by up-regulated proteins, and AKT1S1, CASP8 and SDC1 represent three primary hubs formed by down-regulated proteins (Figure 3D). Enrichment analysis showed that several key cancer signaling pathways are activated in T-R cells, with main interaction nodes formed by components of mTOR, Wnt, p53, metabolic and B cell receptor signaling pathways (Figure 3E). A quantitative representation of these proteins is illustrated in the heat map (Figure 3F).

### Quantitative proteome profiling uncovers the activation of mTOR signaling pathway in trastuzumab-resistant cells

**Figure 4:**
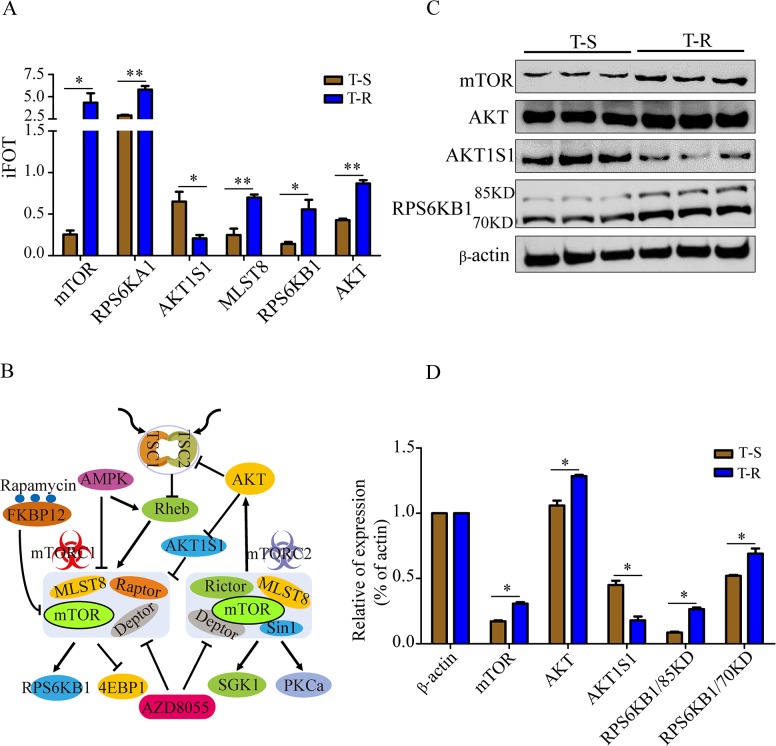
Validation of differentially expressed mTOR pathway proteins **(A)** Six mTOR pathway components that were differentially expressed in T-R and T-S cells measured by mass spectrometry. Data represent mean±SEM of the four independent MS experiments, *p<0.05 and **p<0.01. **(B)** mTOR signaling pathway was illustrated with key molecules highlighted. Arrows represent positive regulation and bald nail show negative regulation between proteins. **(C)** Western blotting analysis of selected mTOR pathway protein expression. Three independent biological replicates were shown. β-actin was used as a loading control. **(D)** Quantification of western blotting signals. Data were shown as mean±SEM of the three independent experiments. *p<0.05.

**Figure 5:**
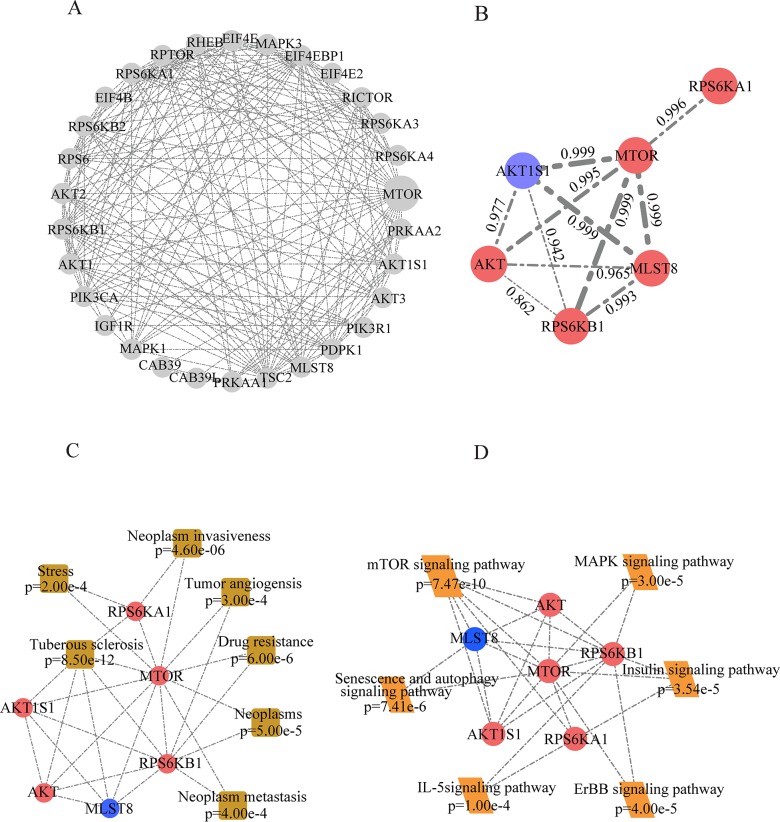
Bioinformatic analysis of differentially expressed proteins involved in mTOR pathway **(A)** Network of all identified proteins associated with mTOR pathway in our dataset. **(B)** Network of differentially expressed proteins in mTOR pathway. Red nodes represent up-regulated proteins and blue nodes represent down-regulated ones. Width of edges and data represent the interaction confidence score. **(C)** Diseases associated with differentially expressed proteins in mTOR pathway based on enrichment analysis in Web Gestalt. **(D)** KEGG analysis identified signaling pathways associated mTOR pathway proteins differentially expressed in T-R cells.

Among the activated pathways in T-R cells, the over-representation of mTOR pathway components caught our attention. MS identified 30 proteins associated with mTOR pathway (Supplementary Table 4_D1), and several key signaling molecules, such as mTOR, AKT, RPS6KB1, RPS6KA1, MLST8 and AKT1S1, showed significant changes in abundance (Figure 4A, Supplementary Table 4_D2). These signaling molecules were represented by a network diagram of mTOR pathway (Figure 4B). Next, we confirmed the altered expression of mTOR, AKT, AKT1S1 and RPS6KB1 by western blotting (Figure 4C). Quantification of the western blot by densitometry revealed significant changes of these proteins between T-S and T-R cells (Figure 4D). As shown in the protein-protein interaction network (Figure 5A), mTOR represents a major hub in the network involving the 30 proteins, and 6 differentially expressed proteins (mTOR, RPS6KB1, AKT, AKT1S1, MLST8 and RPS6KA1) form the core of the network with high interaction confidence scores (Figure 5B). WEB-based Gene Set Analysis Toolkit revealed that these 6 mTOR pathway proteins are associated with multiple pathological conditions. The top 7 cancer relevant processed with the most significant *p* values include tuberous sclerosis, neoplasm invasiveness, drug resistance, neoplasms, neoplasm metastasis, tumor angiogenesis and stress (Figure 5C). Protein-protein interactions derived from the STRING database demonstrated that these proteins participate in several signaling pathways such as mTOR, senescence and autophagy, MAPK, insulin, ErbB and IL-5 pathways (Figure 5D).

### AZD8055 reduces trastuzumab-resistant cells viability, phosphorylation of RPS6KB1 and AKT in T-R cells

**Figure 6:**
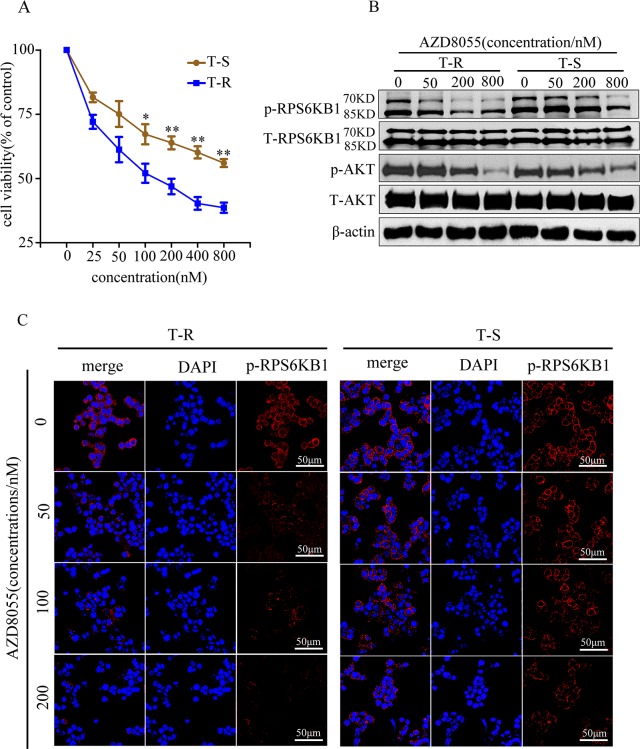
An mTOR inhibitor AZD8055 preferentially inhibited proliferation and phosphorylation levels in NCI N87 T-R cells **(A)** T-S and T-R cells were cultured for 24 h, and then treated with or without AZD8055 at the indicated concentrations (0, 25, 50, 100, 200, 400 and 800 nM) or DMSO as control, respectively. Cell viability was determined by Cell Count kit-8 assay 48 h later. Results expressed as % control represent the mean of three experiments. *p<0.05, **p<0.01 *vs* controls. Error bar represents standard deviations of three replicates. **(B)** Cells were treated for 2 h with AZD8055 at the indicated concentrations (0, 50, 200 and 800 nM), and cell lysates were probed with phosphor- and total antibodies of mTOR signaling pathway. β-actin was used as loading control. Blot shown was representative of at least two independent experiments. **(C)** Cells were treated with AZD8055 at increasing concentrations (0, 50, 100 and 200 mM) for 2 h followed by collection for immunofluorescence. Nuclear staining was performed using DAPI (blue), and *p*-RPS6KB1^(Thr421/Ser424)^ was shown as red points (foci). The representative images were cropped and shown. Original magnification of images ×100.

AZD8055 is a novel ATP-competitive mTOR kinase inhibitor that acts on the PI3K/AKT/mTOR pathway to inhibit cancer cell proliferation and/or induce cancer cell death. It has displayed remarkable ability to target a variety of human cancer cell lines through the inhibition of mTORC1/2 signaling, resulting in a better molecular effect than rapamycin [38–40]. It is currently being assessed, alone or in combination, in phase I clinical trials in oncology [41, 42]. To test the inhibitory effect of AZD8055 on mTOR pathway in GC cells, we treated T-S and T-R cells with increasing concentrations of AZD8055 for 48 h and measured cell viability. AZD8055 suppressed both T-S and T-R cells in a dose-dependent manner, but the effect was more pronounced in T-R cells at all dosages. While the moderate difference was insignificant at 25 and 50 nM (*p*>0.05), it was statistically significant at 100 nM with the cell viability of 67.7% and 52.3% for T-S and T-R, respectively (*p*<0.05) (Figure 6A). A similar trend was observed when cells were treated with higher dosages (*p*<0.01) (Figure 6A). These results showed that T-R cells appeared to be more susceptible to mTOR inhibition by AZD8055 than T-S cells at concentrations above 100 nM, consistent with its stronger dependence on mTOR pathway for survival. To confirm the pathways that mediate trastuzumab resistance, we performed western blotting to examine the phosphorylation levels of RPS6KB1 at Thr421/Ser424 and AKT at Ser473, which are known signaling events in the mTOR pathway. As anticipated, *p*-RPS6KB1^(Thr421/Ser424)^ and *p*-AKT^(Ser473)^ were higher in T-R cells than in T-S cells, and the decrease of *p*-RPS6KB1^(Thr421/Ser424)^ in T-R cells after AZD8055 treatment was more pronounced between 50 and 200 nM, but was not significantly different at 800 nM (Figure 6B). A similar trend was also observed in *p*-AKT^(Ser473)^ (Figure 6B). Moreover, immunofluorescence of *p*-RPS6KB1^(Thr421/Ser424)^ also showed similar results as the western blot (Figure 6C). Taken together, our results strongly suggest that mTOR pathway is activated in T-R cells, which could be preferentially inhibited by AZD8055.

### AZD8055 inhibits the migration and invasion of trastuzumab-resistant cells

**Figure 7:**
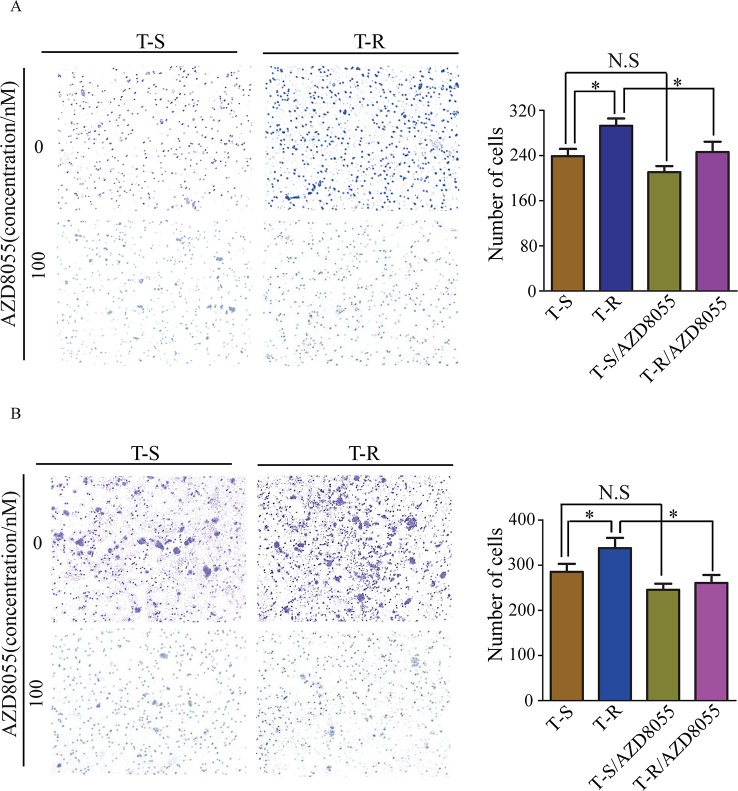
AZD8055 effect on migration and invasion ability of T-S and T-R cells T-S and T-R cells were cultured with or without AZD8055 (100 nM) for indicated times respectively, and then migration and invasion assay were done. **(A**, left panels) Representative image of cell migration and invasion (original magnification × 100). **(B**, right panels) Quantitative results of migration and invasion assays, the results are expressed as mean±SEM of three independent experiments. *p<0.05 compared to controls. N.S: non-significant.

Cancer cells often display high migration and invasion abilities after acquiring drug resistance. Here we evaluated how AZD8055 inhibits migration and invasion of T-S and T-R cells by transwell assays. In the absence of AZD8055, more T-R cells passed through the polycarbonate membrane than T-S cells (*p*<0.05); however, in the presence of AZD8055, migration of T-R cells was inhibited (*p*<0.05), whereas migration of T-S cells was not noticeably inhibited (*p*>0.05) (Figure 7A). Similarly, the higher invasion ability of T-R cells was also significantly inhibited by AZD8055 (*p*<0.05) (Figure 7B). Our results suggest that activation of mTOR pathway promotes migration and invasion *in vitro* in T-R cells, and AZD8055 can preferentially block the mTOR pathway in T-R cells.

## DISCUSSION

HER2 gene amplification and protein over-expression is a driver for malignant transformation, and is associated with poor prognosis and drug resistance [43, 44]. Although trastuzumab represents one of the most successful anti-HER2 antibodies in GC therapy, its therapeutic efficacy is proven to be limited due to acquired drug resistance. While mRNA sequencing has been widely used in identifying transcriptional alterations, increasing evidence suggests that the correlation between protein and mRNA is poor. Clearly if one searches for actionable drug targets, measuring protein is more effective than measuring mRNA. Recent advancement in mass spectrometry instrumentation and method has made proteome profiling a feasible approach. Here, we employed quantitative proteomic profiling strategy to identify pathways that mediate trastuzumab resistance. Our data indicate that multiple mTOR pathway components are expressed at elevated levels in T-R cells, suggesting that mTOR activation plays a crucial role in supporting T-R cell proliferation when HER2 is inhibited.

mTOR is the key component in this pathway and is the target of rapamycin. AKT is the main upstream activator of mTOR, and its activation relieves the inhibitory effects of the TSC1-TSC2 complex on Rheb and mTORC1, thereby activating mTORC1 in response to growth factor stimulations. Activated mTORC1 in turn phosphorylates RPS6KB1, a mediator of protein translation and cell growth. In contrast, AKT1S1, a negative regulator of mTOR, showed a decrease in abundance, suggesting that it may function synergistically with AKT on mTOR activation [45, 46]. Previous studies and current data demonstrate that increased cell proliferation, migration and invasion are characteristics of T-R cells [47, 48]. It is perhaps not surprising that inhibition of mTOR can block the cell migration and invasion, as multiple mTOR effectors are key players in these processes [46, 49] and Rho-Rac signaling is the highest ranking upregulated molecular processes by *p*-value in T-R cells (Figure 3A). It is interesting that, under our experimental conditions, AZD8055 treatment did not seem to cause noticeable blockage of migration and invasion in T-S cells. This result alone does not suggest that mTOR inhibition had no effect for T-S cells, as the dosage and treatment time may need to be optimized. Rather, our results suggest that, among the multiple activated oncogenic pathways, mTOR has become the dominant one that the T-R cells are addicted to for growth and survival, and that targeting this pathway is highly effective.

Our *in vitro* study demonstrated that mTOR inhibition may be considered as a potential treatment strategy for trastuzumab-resistant GC, and mTOR inhibitors in combination with trastuzumab may prevent or delay clinical resistance and improve the efficacy of trastuzumab in GC. Our study also revealed that several other signaling pathways are associated with trastuzumab resistance, which include Wnt pathway, cell cycle, ECM-receptor interaction and metabolic pathway. It has been reported that Wnt pathway can lead to transactivation of EGFR and EMT-like transition, and causes trastuzumab resistance in HER2 overexpressed BC cells [50]. This is consistent with the result presented in a previous study that resistant cells exhibited typical EMT-like phenotype [26]. Admittedly, our exploratory proteomics studies only provide a starting point for a better understanding of the trastuzumab resistance. As the aforementioned pathways can also be targeted for inhibition, a combinatorial therapy targeting multiple pathways can be explored to overcome trastuzumab resistance in the future.

## MATERIALS AND METHODS

### Cell lines and cell culture

Human gastric cancer cells T-S and T-R were kindly provided by the Institute of Basic Medical Sciences Dr. Shi. The cells were cultured in Dulbecco's modified Eagle's Medium (DMEM, Gibco^®^, USA) containing 10% (v/v) fetal bovine serum (FBS) (Gibco^®^, USA), 1% penicillin/streptomycin (100U/mL) at 37°C in a humidified atmosphere with 5% CO_2_. T-R cells were cultured in the same media containing 10μg/mL trastuzumab throughout the study period.

### Chemicals and antibodies

Trastuzumab (Roche Pharmaceutical Ltd., Switzerland) was solubilized in sterile water containing 1.1% benzyl alcohol (stock solution at 100μg/mL, stored at 4°C). Chemicals used are as follows: AZD8055 (Medchem express, USA), dimethyl sulfoxide (DMSO) (Amresco, USA), Dithiothreitol (DTT) and iodoacetamide (IAA) (Sigma-Aldrich, USA), DAPI (Beyotime Biotechnology, China). Cell Count kit-8 was purchased from Dojindo (Kumamoto, Japan). Antibodies against the following proteins were used for immunoblotting: HER-2 (Abcam, UK), mTOR (Abcam, UK), AKT1S1 (Abcam, UK), RPS6KB1 (Cell Signaling Technology, USA), *p*-RPS6KB1 ^(Thr421/Ser424)^ (Cell Signaling Technology, USA), AKT (Cell Signaling Technology, USA), *p*-AKT^(Ser473)^ (Cell Signaling Technology, USA), β-actin (Cell Signaling Technology, USA). Horseradish peroxidase-labeled goat anti-rabbit and anti-mouse secondary antibodies were purchased from ZSGB-BIO (Beijing, China).

### Protein extraction and peptide separation

Cells were lysed in lysis buffer (8M Urea, 100mM Tris Hydrochloride, pH=8.0) containing 1×protease and phosphatase inhibitors (Thermo Fisher Scientific) for 10 min at 0°C, followed by sonication for 1 min (2s on and 2s off, amplitude 25%). The lysates were centrifuged at 16,200 g for 10 min at 4°C, and the supernatants were collected as whole cell extracts (WCE). Protein concentration was determined by Bradford protein assay (ComWin Biotech Co., Ltd, China). One hundred microgram (100μg) protein from each sample was reduced with 10 mM DTT at 56°C for 30 min and alkylated with 10 mM IAA at room temperature in the dark for additional 30 min. Then proteins were digested using the FASP method with trypsin at 37°C in an orbital incubator [51]. Tryptic peptides were separated with a home-made reverse-phase C18 column and eluted using acetonitrile of different percentage as 6%, 9%, 12%, 15%, 18%, 21%, 25%, 30% and 35% (acetonitrile in 10mM NH_4_CO_3_, pH=10). The nine fractions were combined to six fractions (6%+25%, 9%+30%, 12+35%, 15%, 18% and 21%) and dried in a vacuum concentrator.

### LC-MS/MS analysis

An Orbitrap FUSION mass spectrometer (Thermo Fisher Scientific) interfaced with an Easy-nLC 1000 nanoflow LC system (Thermo Fisher Scientific) was used for MS analysis. The dried peptides were dissolved in solvent A (0.1% formic acid in water) and loaded to a C18 reversed-phase column (pre-column: particle size, 3μm, pore size, 120Å, 2cm×100μm, home-made; analytical column: particle size, 1.9μm, pore size, 120Å, 12cm×150μm, homemade) at a flow rate of 600 nL/min for 75 min with a linear gradient of 7∼35% mobile phase B (0.1% formic acid in acetonitrile). The MS full scan was processed in the Orbitrap from m/z 300 to 1,400 with a resolution of 120,000 at 200 m/z. The most intense ions in each scan under top-speed mode were automatically selected in Quadrupole with a 1.6 m/z window and fragmented by higher energy collision-induced dissociation (HCD) with normalized collision energy of 35%, then measured in the linear ion trap. Automatic gain control (AGC) targets were 5e5 ions with a max injection time of 50 ms for full scans and 5e3 with 35ms for MS/MS scans, dynamic exclusion time was employed for 18s. Data were acquired using the Xcalibur software (Thermo Fisher Scientific).

### Protein identification and quantification

The acquired MS/MS spectra were searched by Mascot 2.3 (Matrix Science Inc.) implemented on Proteome Discoverer 2.0 or 2.1 (Thermo Fisher Scientific) against the human National Center for Biotechnology Information (NCBI) Refseq protein databases (updated on 04-07-2013, 32,015 protein entries). The parameter settings were as follows: the mass tolerances were 20 ppm for precursor and 0.5 Da for product ions from FUSION. Up to two missed cleavages were allowed for protease digestion, and the minimal required peptide length was set to seven amino acids. The search engine set cysteine carbamidomethylation as a fixed modification and N-terminal acetylation, oxidation of methionine as dynamic modifications. Precursor ion score charges were limited to +2, +3, +4, +5 and +6. The data were searched against a decoy database so that protein identifications were accepted at FDR of 1%. Label-free protein quantifications were calculated using a label-free, iBAQ approach [52]. A protein's iBAQ divided by the total iBAQ of all identified proteins in one sample was used to define FOT, which was used to estimate the normalized protein abundance and then multiplied by 10^5^ to obtain iFOT5 for the ease of presentation.

### Proteome data filtering and statistical analysis

The proteins that were used for further statistical analysis had to be detected in at least 4 of 8 experiments. The differentially expressed proteins were selected by the following rules: protein abundance ratio ≥ 2, or ≤0.5 for up- or down-regulated, respectively, and *p* value <0.05 using paired two-tailed student test. GO term enrichment analysis was done using WebGestalt website. All identified proteins were defined as background. Protein-protein interaction network analysis was performed using the STRING with interaction sources from experiments and databases and the interaction score set to high confidence (scores > 0.7), the results were displayed using Cytoscape software.

### Cell proliferation assays

Cell proliferation was measured with Cell Count kit-8 in accordance with the manufacturer's instructions. In brief, cells were seeded into 96-well plates in 100μL culture medium at a density of 5,000 cells/well and incubated for 24 h at 37°C. Cells were subsequently incubated with or without AZD8055 at the indicated concentrations (0, 25, 50, 100, 200, 400 and 800 nM) with appropriate vehicle control (DMSO) as a substrate. At 48 h post-treatment, 10% Cell Count kit-8 was added to each well, and the cells were further incubated for 3 h at 37°C. Absorbance was measured at 450 nm using an iMark microplate reader (Bio-Rad, USA). All experiments were done in triplicates and repeated three times.

### Western blotting

Cells were harvested at 70∼80% confluence, and washed twice with cool PBS. Whole cell lysates were extracted using 8M urea containing 1×protease and phosphatase inhibitor for 10 min at 0°C. Lysates were clarified by centrifugation at 16,200g for 20 min at 4°C, and the supernatants were collected as WCE. Protein concentration was determined by Bradford protein assay. A total of 20μg proteins were boiled for five minutes in Laemmli buffer and resolved by SDS-PAGE. Proteins were transferred onto nitrocellulose membrane and blocked with 5% skim milk in TBS-T (10 mM Tris, 150 mM NaCl, 0.05% Tween 20, pH7.4) for one hour. The membrane was incubated overnight at 4°C with primary antibodies followed by TBS-T wash for 5 times, and incubated with horseradish peroxidase-labeled secondary antibody for one hour at room temperature. Proteins were visualized with enhanced chemiluminescence detection reagent.

### Cell migration and invasion assays

Migration and invasion assays were performed using 24-well Transwell^TM^ plates containing polycarbonate filters with an 8-μm pore size (Coring Inc., USA). Five hundred microliter (500μL) of complete DMEM medium was then placed in the lower chamber in the presence or absence of AZD8055 (100nM) or 0.1% DMSO (as control), and 2×10^4^ cells/well suspended in 200μL DMEM without FBS were seeded into the upper chamber. Cells were incubated in a humidified incubator at 37°C for 16 h (for migration assay) or 24 h (for invasion assay), respectively. Un-migrated cells on the upper surface of the membrane were scraped off with a cotton swab, and the migrated cells adhering to the underside of the insert were fixed with formaldehyde for 20 min, stained with 0.1% crystal violet for 20 min at room temperature and then visualized under a microscope (OLYMPUS, BX53, Japan). Cells in six independent symmetrical visual fields were counted at 100 × magnification for quantification of the migration potential. For the invasion assay, the upper chamber was inserted with membranes coated with 80μL Matrigel-matrix^TM^ (BD Discovery Labware, USA), and the bottom one was filled with DMEM containing 10% FBS. Cells on the membranes of inserts were fixed, stained, photographed and counted similarly to the migration assay. Each experiment was performed in three replicates.

### Immunofluorescence analysis

Immunofluorescence analysis was performed after 24 h cell culture. Cells were washed 3 times in PBS containing 1‰ tween-20 for 5min, and fixed in 4% paraformaldehyde for 20 min and permeabilized in 0.5% Triton X-100 for 20 min at room temperature. Cells were washed 3 times and blocked in 1% BSA for one hour. Subsequently, cells were incubated with primary antibody overnight at 4^°^C. After washing, cells were incubated with secondary anti-rabbit IgG antibody conjugated with Alexa 594 (ZSGB-BIO, China) for 30 min at 37°C. Nuclei were stained with 1μg/mL DAPI (4′,6-diamidino-2-phenylindole) for 5 min. Finally, cells were visualized under a laser-scanning confocal microscope (Nikon Ti-E, Japan) for image acquisition. The experiments were repeated in duplicate.

## SUPPLEMENTARY MATERIALS FIGURES AND TABLES











## Abbreviations

T-R: Trastuzumab-resistant
T-S: Trastuzumab-sensitive
GC: gastric cancer
BC: breast cancer
mTOR: mammalian target of rapamycin
LC-MS/MS: liquid chromatography-tandem mass spectrometry
MS: mass spectrometry
WCE: whole cell extracts
FDR: false discovery rate
iBAQ: intensity based absolute quantification
FOT: fraction of total
LFQ: label-free quantification
PCA: principal component analysis
GO: gene ontology
EMT: epithelial-mesenchymal transition
