# Supplementary material for: Quantitative proteomics profiling reveals activation of mTOR pathway in trastuzumab resistance
Source: Oncotarget. 2017 Apr 25;8(28):45793–806. doi: 10.18632/oncotarget.17415 (PMC5542228; doi:10.18632/oncotarget.17415)
Supplement: Supplementary file 1 [file oncotarget-08-45793-s001.pdf]

Quantitative proteomics profiling reveals activation of mTOR pathway in trastuzumab-resistance

SUPPLEMENTARY MATERIALS

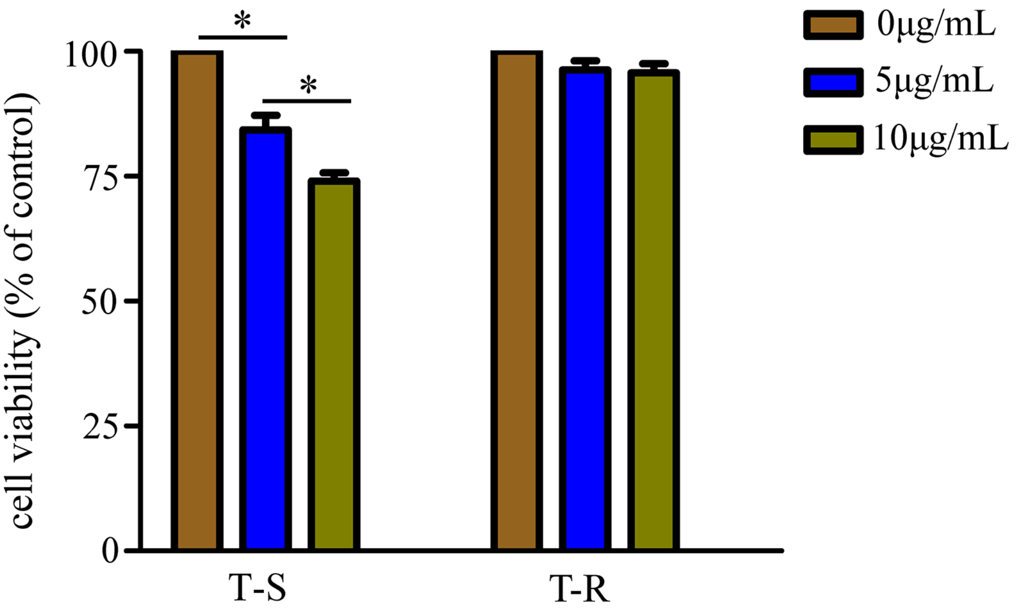

Supplementary Figure 1: Resistance of cells to Trastuzumab evaluated by CCK-8 kit *in vivo*. T-S and T-R cells were cultured in 96-well plates for 4 days with an initial density of 4,000 cells/well in DMEM containing Trastuzumab of different concentration (0, 5 and 10 µg/mL). Data were presented as mean±SEM of three independent experiments. ** $p<0.01$ vs. control.

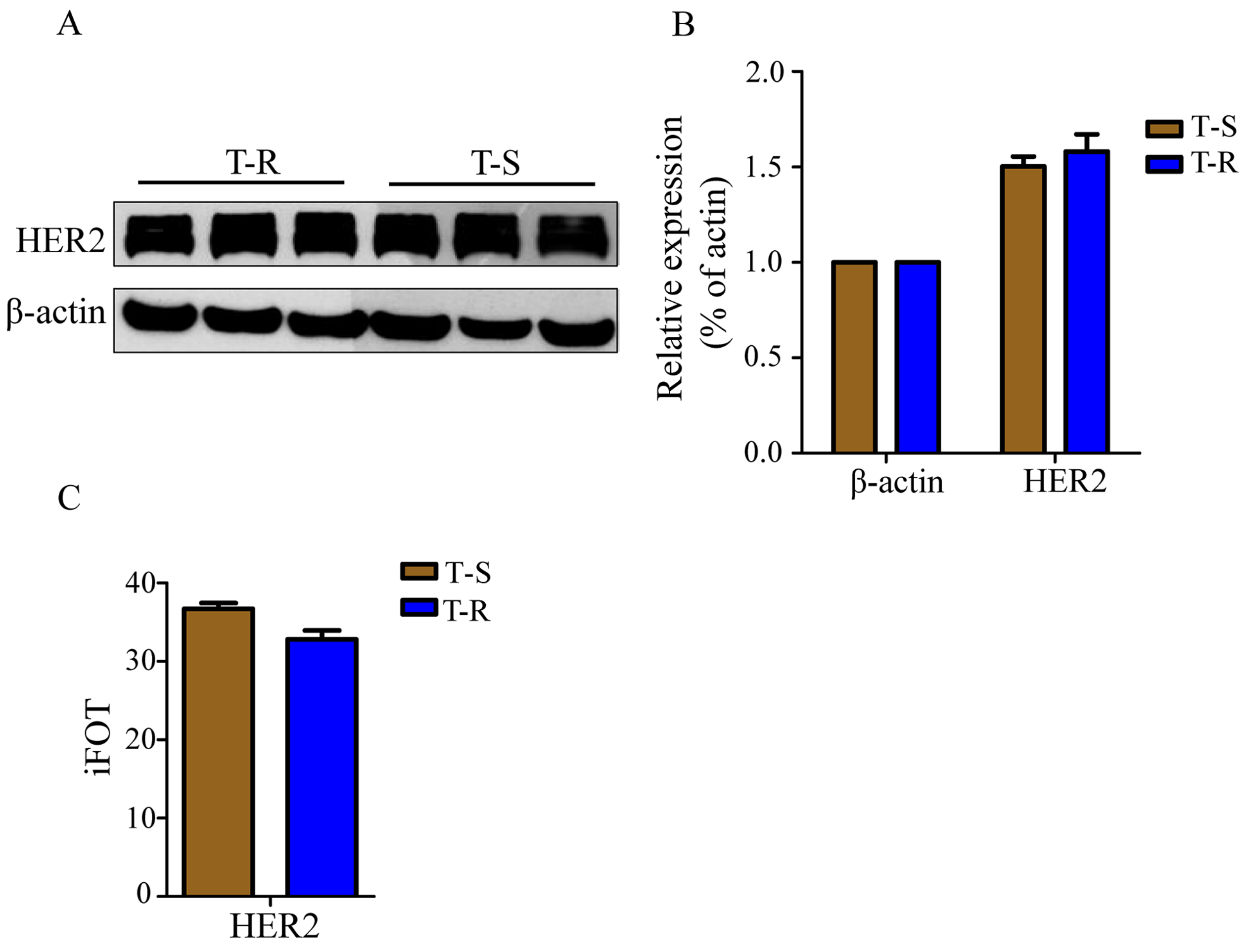

Supplementary Figure 2: (A) Expression of HER2 in T-S and T-R cells was confirmed by western blotting, three independent biological replicates were shown. β-actin was used as a loading plot. **(B)** Quantification of the western blotting signals. Each value presented the mean±SEM of three independent experiments. **(C)** Bar plot showed the expression of HER2 in both cells by MS profiling. Data were presented as mean±SEM of 4 independent experiments.

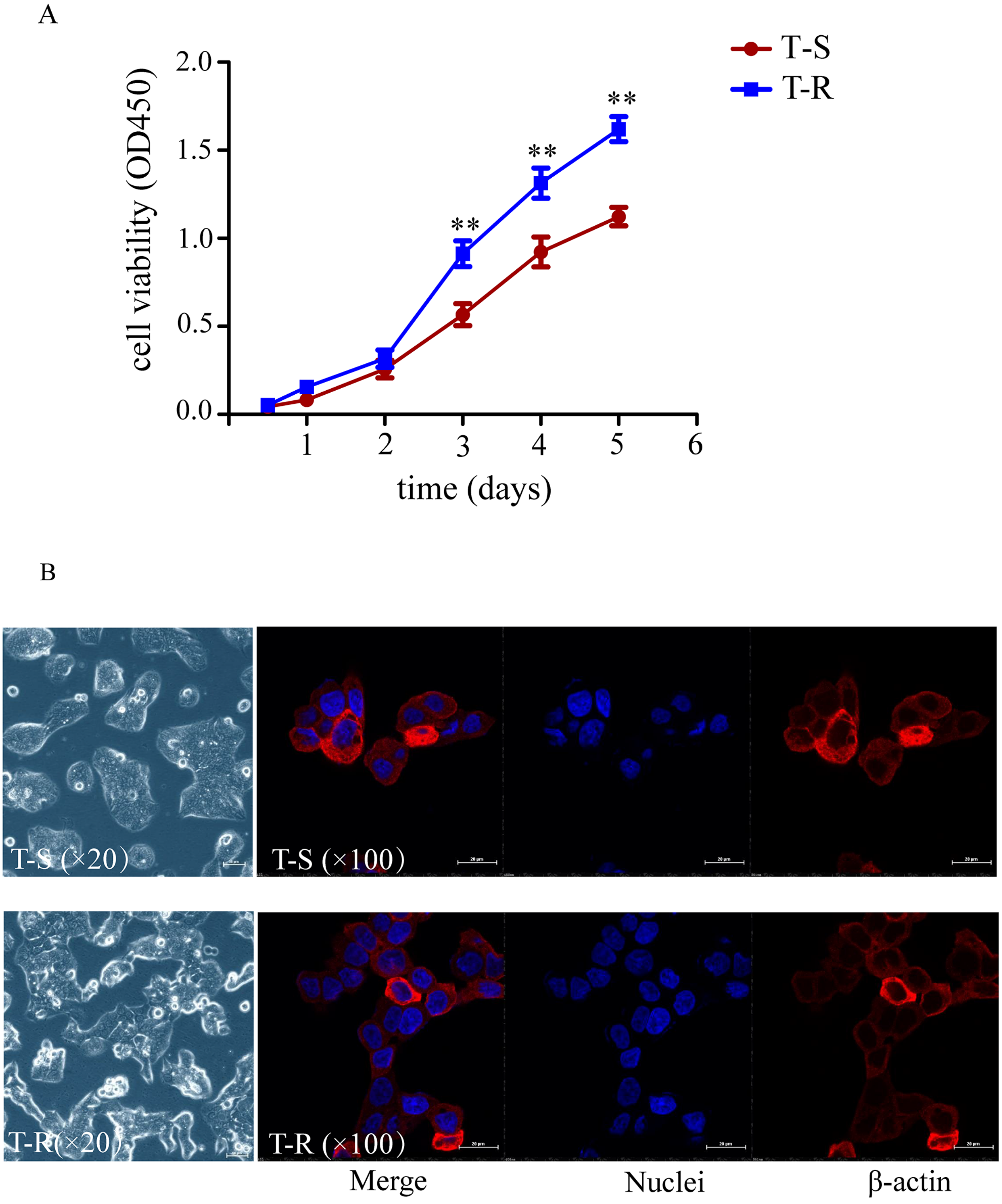

Supplementary Figure 3: (A) Growth curves of T-S cells and T-R cells determined by data from CCK8**.** Data were presented as mean±SEM, n=3. **p< 0.01. **(B)** T-R cells showed morphological changes (image below) comparing with T-S (image above). Cells were observed under a microscope (magnification ×20) and a laser scanning confocal microscope (magnification ×100). Bar=20 µm. Cells were stained with β-tublin to show the morphological changes and tublin filament reorganization (red). DAPI staining was used to show nuclei (blue).

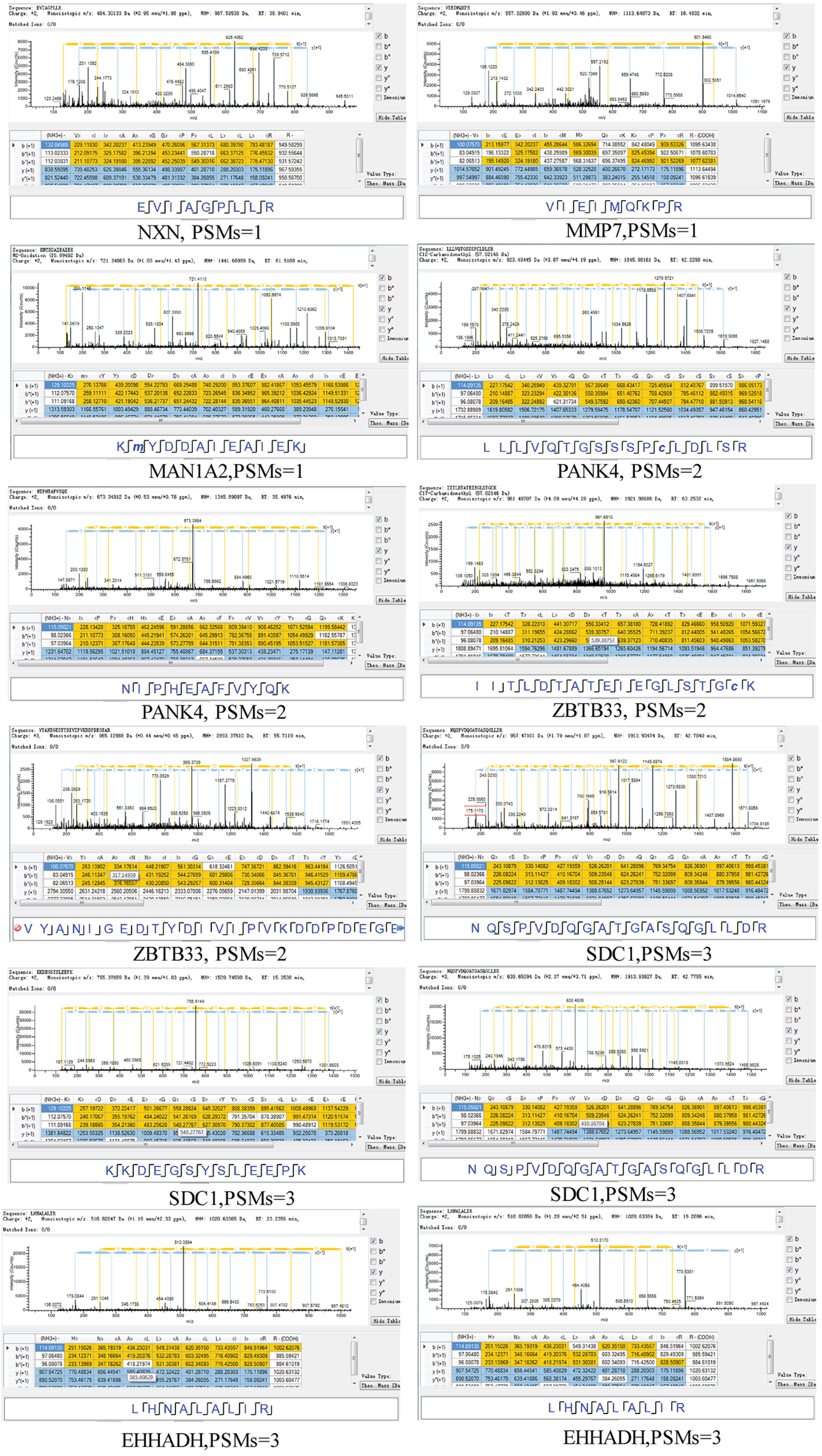

Supplementary Figure 4: Seven differentially expressed proteins identified with minimum numbers spectrum counts (PSMs=1~3) by manual validation.

Supplementary Table 1: All proteins identified and quantified in four replicates at 1% protein FDR at least 2 unique and high quality peptides. All FOT showed are multiplied by 10^5.

See Supplementary File 1

Supplementary Table 2: All proteins identified in at least 4 out of 8 experiments.

See Supplementary File 2

Supplementary Table 3: All differential expressed proteins.

See Supplementary File 3

Supplementary Table 4_D1: All proteins related to mTOR pathway
Supplementary Table 4_D2: Differentially expressed proteins related to mTOR pathway and their values

See Supplementary File 4
